# Pulmonary function in patients with Huntington’s Disease

**DOI:** 10.1186/1471-2466-14-89

**Published:** 2014-05-26

**Authors:** Alvaro Reyes, Travis Cruickshank, Mel Ziman, Kazunori Nosaka

**Affiliations:** 1 School of Medical Sciences Edith Cowan University, 270 Joondalup Drive, Joondalup, 6027, WA, Australia; 2 School of Pathology and Laboratory Medicine, University of Western Australia, Crawley, WA, Australia; 3 School of Exercise and Health Sciences, Centre for Exercise and Sports Science Research, Edith Cowan University, Joondalup, WA, Australia

**Keywords:** Pulmonary function, Huntington’s disease

## Abstract

**Background:**

Huntington’s disease (HD) is a neurodegenerative disorder characterized by progressive motor, cognitive and psychiatric disturbances. Chest muscle rigidity, respiratory muscle weakness, difficulty in clearing airway secretions and swallowing abnormalities have been described in patients with neurodegenerative disorders including HD. However limited information is available regarding respiratory function in HD patients. The purpose of this study was to investigate pulmonary function of patients with HD in comparison to healthy volunteers, and its association with motor severity.

**Methods:**

Pulmonary function measures were taken from 18 (11 male, 7 female) manifest HD patients (53 ± 10 years), and 18 (10 male, 8 female) healthy volunteers (52 ± 11 years) with similar anthropometric and life-style characteristics to the recruited HD patients. Motor severity was quantified by the Unified Huntington’s Disease Rating Scale-Total Motor Score (UHDRS-TMS). Maximum respiratory pressure was measured on 3 separate days with a week interval to assess test-retest reliability.

**Results:**

The test-retest reliability of maximum inspiratory and expiratory pressure measurements was acceptable for both HD patient and control groups (ICC ≥0.92), but the values over 3 days were more variable in the HD group (CV < 11.1%) than in the control group (CV < 7.6%). The HD group showed lower respiratory pressure, forced vital capacity, peak expiratory flow and maximum voluntary ventilation than the control group (p < 0.05). Forced vital capacity, maximum voluntary ventilation and maximum respiratory pressures were negatively (r = -0.57; -0.71) correlated with the UHDRS-TMS (p < 0.05).

**Conclusion:**

Pulmonary function is decreased in manifest HD patients, and the magnitude of the decrease is associated with motor severity.

## Background

Huntington’s disease (HD) is a degenerative disorder of the nervous system characterized by progressive motor, cognitive and psychiatric disturbances [1,2]. Motor abnormalities are caused by specific loss of medium spiny striatal neurons that are responsible for the control, initiation and execution of muscle movements [3]. Respiratory problems such as chest muscle rigidity, respiratory muscle weakness, difficulty in clearing airway secretions and swallowing abnormalities have been reported in patients suffering from neurodegenerative disorders including HD [4,5], but little information is available regarding pulmonary function in HD patients. To the best of authors’ knowledge, only one study reported that pulmonary function was reduced at mid and later stages of HD, however the study was published in an abstract form only [6].

Most of the patients with HD do not report respiratory symptoms until later stages of the disease when the impaired motor control of swallowing muscles and respiratory muscle weakness increase the risk of pneumonia by aspiration, causing death in the majority of patients with HD [3,7-9]. Presumably pulmonary function disturbances remain unnoticed because patients suffering from HD tend to adopt a sedentary life style, with limited physical activity in which impairment of pulmonary function would be evident [10].

The level of pulmonary function impairments in manifest HD patients has not been described in detail to date, and no previous studies have assessed the association between pulmonary function and the severity of motor abnormalities in HD patients. It was hypothesized that pulmonary function would be lower in manifest HD patients in comparison to age-matched healthy volunteers, and the magnitude of the pulmonary deficiency would be associated with an increased level of motor abnormalities of the patients. The present study compared pulmonary function in patients with HD with that of healthy volunteers, and examined the association between pulmonary function and severity of motor abnormalities in manifest HD patients.

## Methods

### Participants

To determine the number of participants required for this study, we performed a pilot study in which spirometry, maximum expiratory (MEP) and inspiratory pressures (MIP) were compared between manifest HD patients and age-matched healthy volunteers. Given that in HD, the main cause of death is an aspiration pneumonia event that is associated with an inability to clear airway secretions in which expiratory muscles have a major role, [11] the sample size calculation was based on MEP. It was estimated that there would be a difference of at least 30 cmH_2_O (SD = 30) in MEP between HD patients and healthy volunteers. With alpha 0.05 and 80% power, an adequate sample size was calculated to be at least 13 participants per group.

Eighteen patients with manifest HD (11 men, 7 women) were recruited using the Huntington’s Enrichment Research Optimisation scheme database [12], with assistance from the Huntington’s Western Australia Association. Inclusion criteria were a positive genetic test, clinically verified disease expression (Unified Huntington’s Disease Rating Scale–Total Motor Score [UHDRS-TMS] ≥5), and the ability to understand and respond to instructions. Exclusion criteria were other confounding neurological disorders, current smokers, occupational or ambient exposure to pollutants that could have affected their pulmonary function, patients with a history of cardiovascular pathology, lung disease, chronic obstructive pulmonary disease (COPD) or the presence of respiratory symptoms such as cough, phlegm, wheezing or dyspnoea at the time of assessment. Patients with HD at later stages of the disease were not considered for this study due to their incapacity to perform a satisfactory pulmonary function tests. The control group (10 men, 8 women) consisted of mainly partners of the recruited HD patients, and some people with similar life-style characteristics to the recruited HD patients. Healthy volunteers were age and gender-matched with the recruited HD patients. The same exclusion criteria as those for the HD patients were applied to the control group. Nine patients with HD and 6 healthy volunteers had a previous history of smoking. Informed written consent was obtained from participants and the study was approved by the Human Research Ethics Committee of Edith Cowan University. Characteristics of the participants are presented in Table 1.

**Table 1:** Physical and physiological characteristics (mean ± SD and ranges) of Huntington’s disease patients (HD) and healthy volunteers (Control)

	**HD**	**Control**	***p***
CAG repeat	43.7 ± 2.6	-	-
	(40–48)		
Disease Burden Score	421.6 ± 92.7	-	-
	(284–615)		
Illness duration (years)	5.4 ± 2.3	-	-
	(3.3-10.2)		
UHDRS-TMS	40 ± 15.7	-	-
	(13 – 62)		
Male/Female	11/7	10/8	-
Age (years)	53 ± 10	52 ± 11	0.359
	(32 – 71)	(37 – 74)	
Height (m)	1.70 ± 0.06	1.70 ± 0.09	0.479
	(1.60 – 1.80)	(1.51 – 1.88)	
Weight (kg)	74.5 ± 15.0	78.5 ± 14.5	0.209
	(56 – 106)	(55 – 110)	
BMI (kg/m^2^)	26.0 ± 4.6	27.0 ± 3.9	0.177
	(20.2 – 36.1)	(21.5 – 35.1)	
Smoking history of ex-smokers* (pack-year)	19.1 ± 31.1	18.7 ± 6.1	0.487
	(0.1 – 92.5)	(7.5 – 26.0)	
Time elapsed from smoke cessation of ex-smokers* (years)	24.4 ± 14.0	20.2 ± 10.1	0.267
	(8 – 50)	(4 – 34)	

P values were based on independent t-tests.

*CAG*: Cytosine-adenine-guanine, *BMI*: Body mass index. *UHDRS-TMS*: Unified Huntington’s disease Rating Scale-Total Motor Score. Pack-year: average daily number of cigarettes smoked divided by 20 and multiplied by the number of years of smoking. *The number of ex-smokers was 9 for HD group, and 6 for control group.

### Maximum respiratory pressure measurement

For MIP and MEP measurements, each participant was asked to sit upright with a nose clip in place to prevent nasal air leakage. A flanged rubber mouthpiece was connected to a pressure manometer (Micro RPM, Micro Medical-Care Fusion, Kent, United Kingdom) and placed in the mouth. Participants were asked to hold the pressure manometer with both hands and to create a tight lip seal around the flanged mouthpiece. A flanged mouthpiece was used as recommended by the American Thoracic Society/European Respiratory Society (ATS/ERS), because it ensures the least air leakage at the mouthpiece [13]. This method was used, since the portability of the manometer is advantageous for clinical use and its reliability has been assessed [14].

For MEP assessment, participants were asked to breathe in to total lung capacity and then to blow hard into the mouthpiece. For MIP assessment, participants were instructed to breath out to residual volume and then to breath in with maximum effort through the mouthpiece. Inspiratory and expiratory efforts were required to be maintained for more than one second. The order of the procedures were first the inspiratory followed by the expiratory effort [13]. Both manoeuvres were repeated a minimum of 5 times with a 30-s rest between measures to minimize the effects of fatigue until three trials showed values within 5% variation of each other. The best result from the three respiratory manoeuvres was used for further analysis as described by Black and Hyatt [15] and the ATS/ERS guidelines [16]. Results of MIP and MEP are expressed as absolute values, because no suitable predictive equations are available.

### Spirometry

Spirometric assessment was performed using a spirometer (Medgraphics, model CPFS/D, St. Paul MN, USA) connected to a laptop computer (Dell, Latitude E6510, USA). The spirometer met all the quality control requirements of the ATS and was calibrated before each testing session with a Hans Rudolph 3.0 syringe, based on the manufacturer’s recommendations. In accordance with the ATS/ERS guidelines [17], each participant was asked to sit upright with a nose clip in place to prevent nasal air leakage. Participants were instructed to perform a slow vital capacity (SVC), a forced vital capacity (FVC) and a maximal voluntary ventilation (MVV) manoeuvre. From these measurements, forced expiratory volume in one second (FEV_1_), peak expiratory flow (PEF) and the ratio of FEV_1_ to FVC (FEV_1_/FVC) from the largest FEV1 and FVC were calculated. Predicted values were calculated for all participants using Stanojevic 2009 [18] prediction equations, which include age, height, weight and gender. The equations include reference values for FVC and FEV_1_, and have been validated for Caucasian Australasian population [19]. Peak expiratory flow predicted values were calculated using Nunn and Gregg [20] regression equations. Given that none of the participants in the study had a confirmed diagnosis of COPD or presented with any known symptoms of COPD, such as cough, sputum production or dyspnea at the time of the assessment, a reversibility test with bronchodilator was not performed.

### Experimental procedures

All the participants of the study were interviewed and completed a general health questionnaire, in which they reported their cardio-vascular and smoking history as well as their respiratory condition. The body mass and height were recorded for each participant using an accurate and calibrated scale (HW200, A&D Mercury Pty, Ltd, Thebarton, SA) and a wall-mounted stadiometer (Model 220, SECA, Hamburg, Germany), followed by MIP and MEP measurements performed over 3 testing sessions with a week apart between sessions.

Thorough explanation, demonstration and practice were provided before attempts at MIP, MEP and spirometric measures. Each participant was instructed to perform spirometric maneuvers in the following order: SVC, FVC and MVV. Each manoeuvre was repeated at least 3 and maximum of 8 times, with 1–2 min rest between attempts. The best result from the three technically acceptable manoeuvres was used for further analysis. For those participants who had difficulties achieving acceptable and reproducible spirograms on the first testing day, the measurement was repeated on different days, to ensure an accurate spirometry. The weekly measurements were taken at the same place and time of day for each participant.

### Statistical analysis

Descriptive data are given as mean and standard deviation. Normality assumption for all continuous variables was tested using the Shapiro-Wilk test. To assess the test-retest reliability of MIP and MEP measurements for each group (HD, control), the interclass correlation coefficient (ICC) and the coefficient of variation (CV) were calculated. To compare spirometric variables, MIP and MEP between HD patients and healthy volunteers, an independent t-test was performed. Relationships between each pulmonary function measurement and UHDRS-TMS were analyzed by a Pearson correlation coefficient. Statistical significance was set at p ≤ 0.05. All statistical analyses were performed using STATA version 9.1.

## Results

There were no significant differences between HD and control groups for gender balance, age, height, body mass and body mass index (Table 1). There were no significant differences in smoking history (p = 0.487) or in the time elapsed since smoking cessation (p = 0.267) between groups (Table 1). A subgroup analysis in HD patients revealed no significant differences in pulmonary function variables between non-smoker (n = 9) and ex-smoker (n = 9) patients. A Shapiro-Wilk test showed that the normality assumption was met for all variables including the variables in the subgroups.

Table 2 shows mean ± SD and range values of MIP and MEP obtained at three different days for HD and control groups. The test-retest reliability of MIP and MEP measurements were acceptable in both groups with no significant differences between three testing sessions. However, the variability for MIP values across testing sessions was greater for HD (CV 10.8%) than control group (CV 5.6%), and this was also the case for MEP (HD: CV 11.1%, control: CV 7.6%). The variability in MIP and MEP was lowest for the second and third testing days for both groups when compared with that for the first and second days or across three days.

**Table 2:** Maximum inspiratory (MIP) and expiratory pressure (MEP) values (mean ± SD and [95% confidence interval]) of Huntington’s disease patients (HD) and healthy volunteers (Control) for 3 different days (Day 1 – Day 3), Interclass correlation coefficient (ICC) for the three measures on 3 different days, and coefficient of variation (CV) for Days 1–3, Days 1 and 2, and Days 2 and 3

		**Day 1**	**Day 2**	**Day 3**	**ICC [95% CI]**	**CV (%) Day 1-2-3**	**CV (%) Day 1-2**	**CV (%) Day 2-3**
MIP	HD	64.2 ± 27.9	67.6 ± 32.4	68.6 ± 30.2	0.94	10.8	12.8	9.2
		[50.3 – 78.1]	[51.5 – 83.7]	[53.6 – 83.6]	[0.89 – 0.98]			
	Control	102.6 ± 27.7	106.8 ± 29.8	106.2 ± 28.5	0.95	5.6	6.0	5.2
		[88.8 – 116.3]	[92.0 – 121.7]	[92.0 – 120.4]	[0.92 – 0.99]			
MEP	HD	94.7 ± 37.6	97.2 ± 36.8	98.5 ± 40.1	0.92	11.1	12.8	9.0
		[76.0 – 113.4]	[78.3 – 118.8]	[78.9 – 115.5]	[0.86 – 0.98]			
	Control	137.8 ± 33.4	142.2 ± 40.6	142.2 ± 40.8	0.92	7.6	7.9	4.8
		[121.1 – 154.4]	[122.0 – 162.4]	121.8 – 162.5]	[0.86 – 0.98]			

As shown in Table 3, the HD group showed significantly lower maximum respiratory pressures, FVC, FEV_1_, PEF and MVV compared with the control group for both absolute and percentage of predicted values. The HD group also showed lower inspiratory capacity (IC) and FEV_1_ than the control group for percentage of predicted values. The FEV_1_/FVC ratio was significantly greater in the HD group compared to the control group. Five participants in the control group and none in the HD group had a FEV_1_/FVC ratio ≤0.7, these 5 participants in the control group had a FEV_1_ (% predicted) greater than 0.7. One of the 5 participants with FEV_1_/FVC ≤0.7 was an ex-smoker. Eight participants in the HD group and 1 in the control presented a FVC (% predicted) equal or lower than 70%. There was no significant difference in SVC between groups.

**Table 3:** Mean ± SD and 95% confidence interval (shown in [ ]) of each pulmonary function variable (absolute and/or percentage predicted values: % predicted) for Huntington’s disease patients (HD) and healthy volunteers (Control)

	**HD**	**Control**	***p***
MIP (cm H_2_O)	72.6 ± 30 [57.7 – 87.6]	110.1 ± 28.8 [95.8 – 124.4]	0.000
MEP (cm H_2_O)	106.2 ± 37 [87.8 – 124.5]	147.9 ± 38.4 [128.8 – 167.0]	0.001
IC (L) % predicted	2.7 ± 0.8 [2.3 – 3.1]	3.2 ± 0.8 [2.8 – 3.6]	0.063
	97.0 ± 24.9 [84.7 – 109.4]	112.3 ± 14.2 [105.2 – 119.4]	0.015
SVC (L) % predicted	3.8 ± 1 [3.3 – 4.4]	4.0 ± 1 [3.6 – 4.6]	0.237
	92.2 ± 23.6 [80.4 – 103.9]	97.4 ± 11.4 [91.7 – 103.0]	0.203
FVC (L) % predicted	3.1 ± 1.0 [2.6 – 3.6]	3.9 ± 1.0 [3.4 – 4.5]	0.008
	72.8 ± 17.4 [64.2 – 81.5]	93.4 ± 13.4 [86.7 – 100.0]	0.000
FEV_1_ (L/s) % predicted	2.6 ± 0.74 [2.2 – 3.0]	3.0 ± 0.72[2.7 – 3.3]	0.066
	80.6 ± 16.8 [72.2 – 89.0]	92.4 ± 12.6 [86.1 – 98.6]	0.011
FEV_1_/FVC	87 ± 7.4 [83.3 – 90.7]	77 ± 8.1 [73.3 – 81.4]	0.000
PEF (L/s) % predicted	5.9 ± 2.0 [4.9 – 6.9]	7.3 ± 1.9 [6.3 – 8.2]	0.024
	64.7 ± 18.4 [55.5 – 73.8]	81.1 ± 18.9 [71.7 – 90.5]	0.006
MVV (L/min) % predicted	103.9 ± 43.0 [82.6 – 125.3]	142 ± 36.6 [124.7 – 161.1]	0.003
	82.6 ± 27.3 [69.0 – 96.1]	117.1 ± 15.0 [109.5 – 124.5]	0.000

*MIP*: Maximum inspiratory pressure, *MEP*: Maximum expiratory pressure, *IC*: Inspiratory capacity, *SVC*: Slow vital capacity, *FVC*: Forced vital capacity, *FEV*_*1*_: Forced expiratory volume in 1 second, *PEF*: Peak expiratory flow, *MVV*: Maximum voluntary ventilation.

*p* values as the results of the t-tests to compare between groups are included.

**Figure 1:**
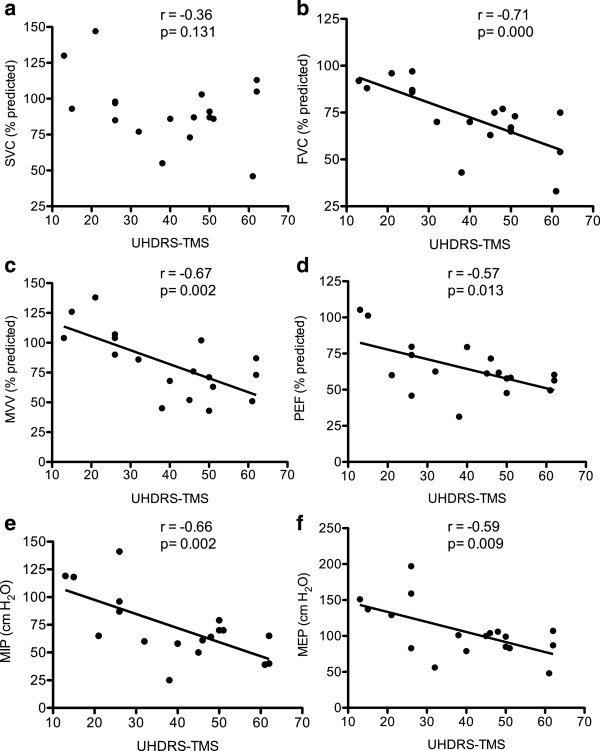
**Correlations between Unified Huntington’s Disease Rating Scale-Total Motor Score (UHDRS-TMS) and slow vital capacity (a), forced vital capacity (b), maximum voluntary ventilation (c), peak expiratory flow (d), maximum inspiratory pressure (e), and maximum expiratory pressure (f).** r values of a Pearson correlation coefficient and p values are included. Spirometric indices are expressed as % of predicted values.

Significant correlations between maximum respiratory pressure and FVC or PEF were found only in the HD group either for percentage of predicted values (Table 4) or absolute values. Figure 1 shows relationships between the UHDRS-TMS and pulmonary function variables (percentage of predicted values). All of the variables except SVC were negatively correlated with the UHDRS-TMS such that the greater the UHDRS-TMS, the smaller the variables (Figure 1).

**Table 4:** **Correlation between maximum inspiratory pressures (MIP, absolute values) or maximum expiratory pressure (MEP, absolute value) and pulmonary function variables (inspiratory capacity: IC, slow vital capacity: SVC, forced vital capacity: FVC, forced expiratory volume in 1 second: FEV**_**1**_**, peak expiratory flow: PEF, maximum voluntary ventilation: MVV, percentage predicted values) in Huntington’s disease (HD) and healthy volunteers (Control)**

			**IC (% predicted)**	**SVC (% predicted)**	**FVC (% predicted)**	**FEV**_**1**_**(% predicted)**	**PEF (% predicted)**	**MVV (% predicted)**
MIP (cm H_2_O)	HD	*r*	0.26	0.40	0.75	0.68	0.69	0.56
		*p*	0.293	0.091	0.000	0.001	0.001	0.014
	Control	*r*	0.20	0.14	0.14	0.21	0.16	0.17
		*p*	0.410	0.577	0.558	0.402	0.509	0.479
MEP (cm H_2_O)	HD	*r*	0.18	0.51	0.67	0.64	0.38	0.50
		*p*	0.461	0.028	0.002	0.003	0.113	0.032
	Control	*r*	0.20	0.34	0.27	0.12	0.22	-0.04
		*p*	0.423	0.158	0.262	0.629	0.373	0.848

*r* values of a Pearson correlation coefficient and *p* values are shown.

## Discussion

The results of the present study showed that pulmonary function was reduced in manifest HD patients when compared with the age-matched healthy volunteers (Table 2). Pulmonary function variables were negatively correlated with UHDRS-TMS, suggesting that decreased pulmonary function is associated with the severity of motor abnormalities (Figure 1).

Maximum respiratory pressure values had higher variation in HD patients relative to the control group. Moreover the accuracy of MIP and MEP measurements improved after the first testing session for both groups. These results coincide with those reported in reliability studies of maximal respiratory pressure in patients with multiple sclerosis [21] and healthy volunteers [14]. The results suggest that at least one previous testing session is necessary to achieve more accurate values. It appears that the learning process takes longer in patients suffering from neurological disorders than it does in healthy participants.

The subgroup analysis in HD patients and controls revealed no significant differences in pulmonary function between non-smokers and ex-smokers. As shown previously, spirometry can detect obstructive lung damage after 20 pack-years of smoking [22]. In the present study however, both HD and control groups had mean pack-years of smoking less than 20, and the time elapsed from smoke cessation was at least 20 years on average for HD and control groups. Prospective studies have shown that decreased lung function in smokers without respiratory chronic symptoms normalises after 2 years of smoking cessation [23,24]. Although the subgroup analysis in HD and controls between non-smokers and ex-smokers lacks statistical power, the effects of smoking on the pulmonary function were minimal if any in the present study, and it does not appear that the smoking history was a confounding factor affecting the variables of the pulmonary function. In the control group, the 6 ex-smokers had lower values (percentage of predicted) of SVC, FVC, FEV_1_ and PEF in comparison to those 12 non-smokers, which decreased sprometric indices in the whole control group (Table 3). Although the 6 ex-smokers had values below predicted, these values were within normal ranges. It should be noted that none of the participants of the present study had symptoms of COPD. Thus, the impaired pulmonary function in the HD patients was due to the disease itself.

The differences presented in this study between HD patients and control volunteers (e.g. FVC: -21%. PEF: -16%, MVV: 35%) were similar to those reported in patients with Parkinson’s disease (PD). Sathyaprabha et al. [25] reported significantly decreased MIP (-48%), MEP (-47%) and FVC (-28%) in PD patients compared with a control group. Polatli et al. [10] reported significantly decreased PEF (-20%) and MVV (-37%) in PD patients compared to a control group, and also showed that FVC and MVV (% predicted values) were correlated (r = -0.65 and r = -0.87, respectively) with disease severity in moderate affected PD patients. Analogous pathophysiological features of PD and HD may explain these similar findings between patients with these diseases.

In the present study, MIP exhibited greater positive correlation with PEF than MEP in the group of HD patients (Table 4). Trebbia et al. [26] reported that MIP had a higher correlation with peak cough flow than MEP in patients with neuromuscular disorders. Inspiratory muscle weakness and reduced inspiratory capacity (IC) are important factors limiting cough efficacy. The inspired volume of air that precedes coughing determines the expelled volume of air and the length-tension relationship of the expiratory muscles, thus this length-tension relationship influences the capacity of expiratory muscles to produce force [27,28]. In the present study, IC (percentage of predicted value) was significantly reduced in the HD group when compared with the control group, however IC did not show relevant correlations with PEF or maximum respiratory pressures. This suggests that in patients with HD, MIP contributes more than IC to produce a better PEF. Further studies are needed regarding cough determinants in HD patients.

Motor related manifestations of adult-onset presentation of HD include chorea, bradykinesia, hypokinesia, akinesia, muscle weakness and rigidity [9,29,30]. Choreic movements can affect all parts of the body including respiratory and swallowing muscles [29]. The amplitude and severity of choreic movements vary between individuals. Huntington’s disease patients predominantly show a choreo-athetoid phenotype, while a smaller proportion of individuals present an akinetic/rigid state [2]. As the disease progresses however, choreic movements tend to decrease and akinesia and rigidity become more prominent [29,31]. It has been reported that bradykinesia is greater in patients with the akinetic/rigid phenotype [30,32]. The mechanisms underlying the respiratory problems in neurodegenerative disorders including HD are unknown [5], however motor abnormalities such as bradykinesia, rigidity and respiratory muscle weakness are likely to be associated with them. It is important to note that the pulmonary function parameters related to forced respiration are mainly affected in manifest HD patients.

The negative correlations between pulmonary function variables and UHDRS-TMS (Figure 1) suggest that the greater the movement disorders, the greater the reduction in the performance of repetitive respiratory tasks such as MVV, and the smaller the capacity to generate fast and explosive respiratory muscle contractions required during FVC, PEF and maximum respiratory pressure manoeuvres. Although the results of the present study showed decrements of pulmonary function in the HD patient group, it is important to note that such impairments were not present in all patients. There were some overlaps in pulmonary function results between HD patients and healthy participants, suggesting that the nature and severity of pulmonary complications vary between patients.

Given that pulmonary complications are the major cause of death in patients with HD and that decreases in pulmonary function are associated with greater severity of motor abnormalities, it is important to include pulmonary function tests at early stages of the disease. If pulmonary function impairment is detected, a specific intervention such as respiratory muscle training, manually assisted coughing, or mechanical cough assistance should be implemented to prevent an aspiration pneumonia event. The benefits obtained from these interventions would have a higher impact on HD patients at early stages of the disease when muscles abnormalities are not yet severer than those at late stages when muscle and neural damage become more extensive. How HD patients respond to respiratory muscle training should be investigated in future studies.

## Conclusion

The results of the present study indicate that pulmonary function is decreased in individuals with manifest HD. In addition decreased pulmonary function is associated with greater severity of motor abnormalities. Pulmonary function tests are useful to monitor HD patients from early stages of the disease to prevent future severe respiratory complications.

## Abbreviations

ATS: American thoracic society; ERS: European respiratory society; CV: Coefficient of variation; COPD: Chronic obstructive pulmonary disease; FEV1: Forced expiratory volume in 1 second; FVC: Forced vital capacity; HD: Huntington’s disease; IC: Inspiratory capacity; ICC: Interclass correlation coefficient; MIP: Maximum inspiratory pressure; MEP: Maximum expiratory pressure; MVV: Maximum voluntary ventilation; PD: Parkinson’s disease; PEF: Peak expiratory flow; SVC: Slow vital capacity; UHDRS-TMS: Unified huntington’s disease rating scale total motor score.

## Competing interest

The authors declare that they have no competing interest.

## Authors’ contributions

AR is responsible for the integrity of the work as a whole, from inception to published article. He was also responsible for the conception and design of the study, analysis of the results and writing of the paper. TC made substantial contributions to acquisition and interpretation of data; he critically revised the manuscript for important intellectual content. MZ contributed to interpretation of the results, critical revision of the article for important intellectual content, editing of the paper and final approval of the version to be published. KN contributed to study design, critical revision of the article for important intellectual content, editing of the paper, and final approval of the version to be published. All authors read and approved the final manuscript.

## Pre-publication history

The pre-publication history for this paper can be accessed here:

http://www.biomedcentral.com/1471-2466/14/89/prepub
